# Functional MRI Assessment of Task-Induced Deactivation of the Default Mode Network in Alzheimer’s Disease and At-Risk Older Individuals

**DOI:** 10.3233/BEN-2009-0231

**Published:** 2009-10-21

**Authors:** Maija Pihlajamäki, Reisa A. Sperling

**Affiliations:** ^1^Memory Disorders UnitDepartment of NeurologyBrigham and Women's HospitalHarvard Medical SchoolBostonMAUSA; ^2^Unit of NeurologyInstitute of Clinical MedicineUniversity of KuopioKuopioFinland; ^3^Department of PsychiatryMassachusetts General HospitalBostonMAUSA

**Keywords:** Aging, Alzheimer's disease (AD), apolipoprotein E, default mode network, functional magnetic resonance imaging (fMRI), memory, mild cognitive impairment (MCI), posterior cingulate cortex, precuneus, task-induced deactivation

## Abstract

Alzheimer’s disease (AD) is the most common form of dementia in old age, and is characterized by prominent impairment of episodic memory. Recent functional imaging studies in AD have demonstrated alterations in a distributed network of brain regions supporting memory function, including regions of the default mode network. Previous positron emission tomography studies of older individuals at risk for AD have revealed hypometabolism of association cortical regions similar to the metabolic abnormalities seen in AD patients. In recent functional magnetic resonance imaging (fMRI) studies of AD, corresponding brain default mode regions have also been found to demonstrate an abnormal fMRI task-induced deactivation response pattern. That is, the relative decreases in fMRI signal normally observed in the default mode regions in healthy subjects performing a cognitive task are not seen in AD patients, or may even be reversed to a paradoxical activation response. Our recent studies have revealed alterations in the pattern of deactivation also in elderly individuals at risk for AD by virtue of their APOE e4 genotype, or evidence of mild cognitive impairment (MCI). In agreement with recent reports from other groups, these studies demonstrate that the pattern of fMRI task-induced deactivation is progressively disrupted along the continuum from normal aging to MCI and to clinical AD and more impaired in e4 carriers compared to non-carriers. These findings will be discussed in the context of current literature regarding functional imaging of the default network in AD and at-risk populations.

## 1. Introduction

Alzheimer's disease (AD) is the most common form of dementia in old age, a characteristic feature of which is the difficulty in forming new memories of everyday life-events. Successful formation of long-term episodic memories is a complex cognitive process that is thought to require synchronized interaction of several large-scale neural networks, one of the core networks being linked to the hippocampus and surrounding medial temporal structures [18,49,77]. Recent functional magnetic resonance imaging (fMRI) studies have suggested that the hippocampal memory network is, at least partially, topographically overlapping with the so called brain default mode network [6,7,27,65,76,88]. The default mode network is considered to represent a network of brain regions that typically present with co-

ISSN 0953-4180/09/$17.00 © 2009 – IOS Press and the authors. All rights reserved ordinated low frequency fluctuations and elevated activity in states of relative rest [6,65,76]. The key default mode regions predominantly consist of medial and lateral parietal regions extending into posterior cingulate and retrosplenial cortices as well as midline and lateral frontal regions. These same regions that are activated at rest appear to be suppressed during various cognitive activities, including encoding of new memories [63,70, 76]. Deactivation of key nodes of the default mode network, in coordination with hippocampal activation, may in fact be a prerequisite for focused attention and successful memory encoding [13,50,91]. Conversely, there is evidence that failure to suppress activity in some of the core default mode areas such as the posteromedial cortices is associated with failed encoding and poor performance in subsequent memory tests in cognitively normal young and old subjects [25,50,57].

Predilection of the posteromedial core regions of the default network [21] to demonstrate task-induced deactivation (i.e., relative decreases in the blood-oxygen-level-dependent (BOLD) fMRI signal) during paradigms requiring attention to external stimuli has been consistently demonstrated in young and old healthy subjects using a multitude of different cognitive stimuli and both fMRI and PET imaging modalities [6–8,13,30,46,50,57,65,76]. Consistency of the human brain resting state networks has also been demonstrated using a different methodological approach, that is a tensor probabilistic independent component analysis applied to resting-state fMRI data in healthy young subjects [12]. In line with the studies reporting diminished posteromedial cortical deactivation during unsuccessful encoding in healthy subjects [25,50,57], recent fMRI studies have reported evidence of an abnormal task-induced deactivation pattern in clinical AD patients compared to elderly controls in corresponding posteromedial regions of the default mode network [28, 44,61–63,70,71]. Alterations in the hippocampal – posteromedial memory networks in AD have also been demonstrated using fMRI and independent component analysis [9].

The term "mild cognitive impairment, MCI", and amnestic MCI in particular, is currently the most commonly used term to refer to the clinical risk stage of AD [59]. Most studies suggest that approximately 12–15% of MCI subjects will progress to dementia within one year's follow-up time. MCI is, however, known to be a heterogeneous population, in terms of clinical, imaging and even pathological findings [37]. Similarly, findings from previous memory-fMRI activation studies in MCI subjects have been relatively variable [15, 16,31,39,45]. The main known genetic risk factor for late onset AD is the e4 allele of the apolipoprotein E (*APOE*) gene on chromosome 19 [3,72]. Compared to the most common allele *APOE* e3, the e4 allele increases, whereas e2 decreases, the risk of AD and lowers the mean age at disease onset in a dose-dependent way [19]. So far, fMRI studies investigating the effect of the *APOE* e4 risk genotype [58,64], or the combined effect of MCI and e4, on the fMRI deactivation response pattern are not many and therefore, more studies are needed to complement and corroborate the currently existing findings of altered fMRI task-induced deactivation in elderly subjects at risk for AD [58,61, 62,64,70,71].

Our recent studies have focused on probing fMRI task-induced deactivations in the default network regions in the two common AD at-risk populations, that is, subjects with MCI and/or an *APOE* e4 carrier status [64]. We have also examined whether standard clinical cognitive measures such as the Mini-Mental State Examination (MMSE) and Clinical Dementia Rating Sum-of-Box (CDR-SB) scores and fMRI experimental measures of memory are correlated with the fMRI deactivation pattern during a memory encoding task, across the full spectrum older subjects from healthy elderly individuals to patients with mild AD. In this paper, we will discuss the results of our own studies, and review the current literature regarding the potential diagnostic and prognostic use of functional imaging of the default network in AD and in subjects at risk for AD using various MRI and positron emission tomography (PET) techniques.

## 2. Material and methods

### 2.1. Participants

Seventy-four older individuals participated in these studies. All subjects provided informed consent in accordance with the Human Research Committee guidelines of the Massachusetts General Hospital and Brigham and Women's Hospital (Boston, MA, USA). Subjects (Table 1) were classified into three clinical study groups on the basis of their Clinical Dementia Rating (CDR) scale [54]. Normal older control subjects (OC) ($n = 29$) had a CDR = 0.0. The MCI subjects ($n = 30$) met the following criteria for MCI [60]: 1) a memory complaint corroborated by an informant, 2) normal general cognitive function, 3) normal activities of daily living, 4) an overall CDR of 0.5, and 5) not demented. The AD patients ($n = 15$) met NINCDS-ADRDA criteria for probable AD [47], and had mild dementia severity as characterized by an overall CDR = 1.0. Sixty-four of the participants (OC: $n = 29$; MCI: $n = 30$; AD: $n = 5$) were recruited from a longitudinal study examining preclinical predictors of AD and the remaining ten subjects (AD: $n = 10$) from memory disorder clinics.

The subjects were also divided into subgroups by their *APOE* e4 genotype (Table 1). Among the OC there were 8/29 e4 carriers (28%), among the MCI 10/30 (33%) and among the AD patients 9/15 (60%) e4 carriers. In this study sample, there was only one e4 homozygote, a subject with MCI. The *APOE* polymorphisms were genotyped by restriction fragment length analysis following polymerase chain reaction from $\sim$10 nanograms of genomic DNA, as described previously (Hixson and Vernier, 1990).

### *2.2. fMRI activation task*

The fMRI activation task [81,82] consisted of processing of blocks of Novel and Repeated face-name pairs alternating with simple visual Fixation baseline. For the Fixation, the participants were instructed to focus their attention on a white cross-hair presented on the black background. For the Novel and Repeated activation conditions, they were instructed to try i) to remember the name associated with each face, ii) to make a decision regarding whether or not they thought the name "fit" the face, and iii) to press the response button with their index or middle finger accordingly. The stimuli were arranged in a block design with each fMRI run consisting of three different conditions: two Novel blocks (7 face-name pairs per block, each shown for 5 s) and two Repeated blocks of identical length, separated by 25 s periods of Fixation. The entire functional scanning session included six runs, 4 min 15 s each. Duration of the Novel and Repeated activation blocks together was 140 s (equal to 56 fMRI acquisitions), and duration of the Fixation condition 115 s (equal to 46 fMRI acquisitions) per one run. Visual stimuli were presented using MacStim 2.5 software (WhiteAnt Occasional Publishing, West Melbourne, Australia). Images were projected through a collimating lens onto a screen attached to the head coil. Responses were collected using a fiber-optic response box held in the right hand.

### *2.3. Post-scan memory test*

Approximately 5 min after the scanning session was completed, all subjects underwent two brief memory tests – a face recognition test and a forced-choice name recognition test – which were presented on a computer screen outside of the scanner. The first memory test (MT1) consisted of 12 of the Novel faces presented only once during the experiment, the two Repeated faces, as well as eight faces not seen during the experiment that were presented as foils. Subjects were instructed to indicate whether they had seen the face while in the scanner or not. The second memory test (MT2) was a two-forced-choice associative recognition task during which a different set of twelve Novel faces seen during the experiment and again the two Repeated faces were presented. Each face was shown with two names printed underneath: the correct name that was paired with the face during scanning and one incorrect name that was previously paired with a different face during scanning. The subjects were instructed to indicate the correct name by pointing to it on the computer monitor.

### *2.4. fMRI data acquisition*

Subjects were scanned using a Siemens Trio 3.0 T scanner (Siemens Medical Systems, Iselin, NJ) equipped for echo-planar imaging (EPI) with a 3-axis gradient head coil. High resolution structural images were acquired using a $T_1$-weighted 3D MP-RAGE (Magnetization Prepared Rapid Acquisition Gradient Echo) sequence with the following parameters: repetition time (TR) = 2530 ms, echo time (TE) = 3.45 ms, inversion time (TI) = 1100 ms, flip angle (FA) = 7°, field of view (FOV) = 256 mm, matrix = 192 × 256, slice thickness = 1.33 mm, 128 sagittal slices. BOLD fMRI data were acquired using a $T_2^*$-weighted gradient-echo EPI sequence with the following parameters: TR = 2500 ms, TE = 30 ms, FA = 90°, FOV = 200 mm, matrix = 64 × 64, resulting in an in-plane resolution of 3.125 × 3.125 mm$^2$. Twenty-eight oblique coronal slices with a thickness of 5.0 mm and an interslice gap of 1.0 mm were acquired, oriented perpendicular to the anterior-posterior commissural line. Each functional run consisted of 102 whole-brain acquisitions; the first 4 functional volumes were discarded from the data analysis to allow for $T_1$-stabilization.

Table 1
Characteristics of the clinical groups and genetic subgroups

| | OC ($n = 29$) | | MCI ($n = 30$) | | AD ($n = 15$) | |
|---|---|---|---|---|---|---|
| | OC $\varepsilon$4– ($n = 21$) | OC $\varepsilon$4+ ($n = 8$) | MCI $\varepsilon$4– ($n = 20$) | MCI $\varepsilon$4+ ($n = 10$) | AD $\varepsilon$4– ($n = 6$) | AD $\varepsilon$4+ ($n = 9$) |
| *Demographics* | | | | | | |
| Age (range) | 74.2 ± 5.6 (66–90) | | 76.7 ± 5.3 (67–87) | | 78.3 ± 6.9* (57–85) | |
| Age (range) | 74.8 ± 5.4 (68–90) | 72.6 ± 6.0 (66–81) | 76.8 ± 5.6 (67–87) | 76.5 ± 4.7 (67–84) | 77.0 ± 10.1 (57–84) | 79.1 ± 4.3 (73–85) |
| Female/male; % of female | 19/10; 66 | | 19/11; 63 | | 8/7; 53 | |
| Female/male; % of female | 11/10; 52 | 8/0; 100 | 13/7; 65 | 6/4; 60 | 3/3; 50 | 5/4; 55 |
| *Global cognition* | | | | | | |
| CDR-SB range) | 0.0 ± 0.0 (0.0–0.0) | | 1.8 ± 1.0* (0.5–3.5) | | 4.6 ± 0.7*# (3.5–6.0) | |
| CDR-SB (range) | 0.0 ± 0.0 (0.0–0.0) | 0.0 ± 0.0 (0.0–0.0) | 1.6 ± 1.0 (0.5–3.5) | 2.4 ± 0.8& (0.5–3.5) | 5.2 ± 0.4 (5.0–6.0) | 4.2± 0.5§ (3.5–5.0) |
| MMSE (range) | 29.7 ± 0.5 (29–30) | | 29.1 ± 1.1* (26–30) | | 23.3 ± 4.2*# (15–30) | |
| MMSE (range) | 29.6 ± 0.5 (29–30) | 29.8 ± 0.4 (29–30) | 29.2 ± 1.1 (26–30) | 28.9 ± 1.1 (27–30) | 23.2 ± 5.2 (15–29) | 23.3 ± 3.7 (17–30) |
| *Post-scan memory* | | | | | | |
| Face recognition % (range) | 72.3 ± 12.6 (50–100) | | 76.0 ± 12.7 (55–100) | | 68.5 ± 12.1 (50–91) | |
| Face recognition % (range) | 71.3 ± 11.7 (50–91) | 74.9 ± 15.3 (50–100) | 76.5 ± 11.6 (59–100) | 75.1 ± 15.3 (55–100) | 64.3 ± 10.1 (50–77) | 71.3 ± 13.0 (55–91) |
| Name recognition % (range) | 87.8 ± 9.4 (64–100) | | 84.1 ± 13.0 (43–100) | | 65.7 ± 11.7*# (50–86) | |
| Name recognition % (range) | 86.5 ± 9.9 (64–100) | 91.3 ± 7.2 (79–100) | 86.5 ± 8.7 (71–100) | 79.4 ± 18.6 (43–100) | 68.0± 12.8 (50–79) | 64.2 ± 11.5 (50–86) |

Mean ± standard deviation. AD = Alzheimer's disease; CDR-SB = Clinical Dementia Rating Sum-of-Box score; $\varepsilon$4– = no $\varepsilon$4 alleles; $\varepsilon$4+ = carrier of at least one $\varepsilon$4 allele; MCI = mild cognitive impairment; MMSE = Mini-Mental State Examination; OC = older control. Significant differences between the genetic subgroups ($p < 0.05$, Mann-Whitney U test) are indicated as follows: **vs* OC; #*vs* MCI; &*vs* MCI $\varepsilon$4–; §*vs* AD $\varepsilon$4–.

### 2.5. fMRI data analysis

In this study, we wanted to investigate alterations in fMRI task-induced deactivation responses, (i.e., relative decreases in BOLD signal) in AD patients and in subjects at risk for AD. The contrast to reveal task-induced deactivation was defined as active processing of Novel and Repeated face-name pairs compared to passive Fixation baseline. For the purposes of this study, this contrast was considered the most meaningful, as the cognitive and motor task instruction given to the subjects was the same during both the Novel and Repeated blocks, the amount of visually presented information was the same during the Novel and Repeated slides, and this approach utilized all of the fMRI data from each run.

fMRI data analysis was carried out using FEAT version 5.43, part of FSL (www.fmrib.ox.ac.uk/fsl). The following pre-statistical processing was applied: motion correction, removal of non-brain structures, spatial smoothing using a Gaussian kernel of full width at half maximum of 5 mm, mean-based intensity normalization of all volumes by the same factor, and high-pass temporal filtering with a frequency cutoff point of 140.0 s. Time-series statistical analysis was carried out using FILM with local autocorrelation correction. FMRI images were registered to high resolution structural images and to the Montreal Neurological Institute (MNI) standard brain. Higher-level analysis within and between the study groups was carried out using FLAME [2,93]. The resulting Z-statistic images (Gaussianised T-statistic images) were corrected for multiple comparisons using cluster thresholding [94], applying default FSL values for the voxelwise Z-statistic threshold (Z > 2.3) and for the cluster probability threshold ($p < 0.05$).

Based on the previous observations that AD-related changes in the default network function are observed in the posteromedial nodes of this network in particular [21,28,44,50,67,78], we focused the data analyses

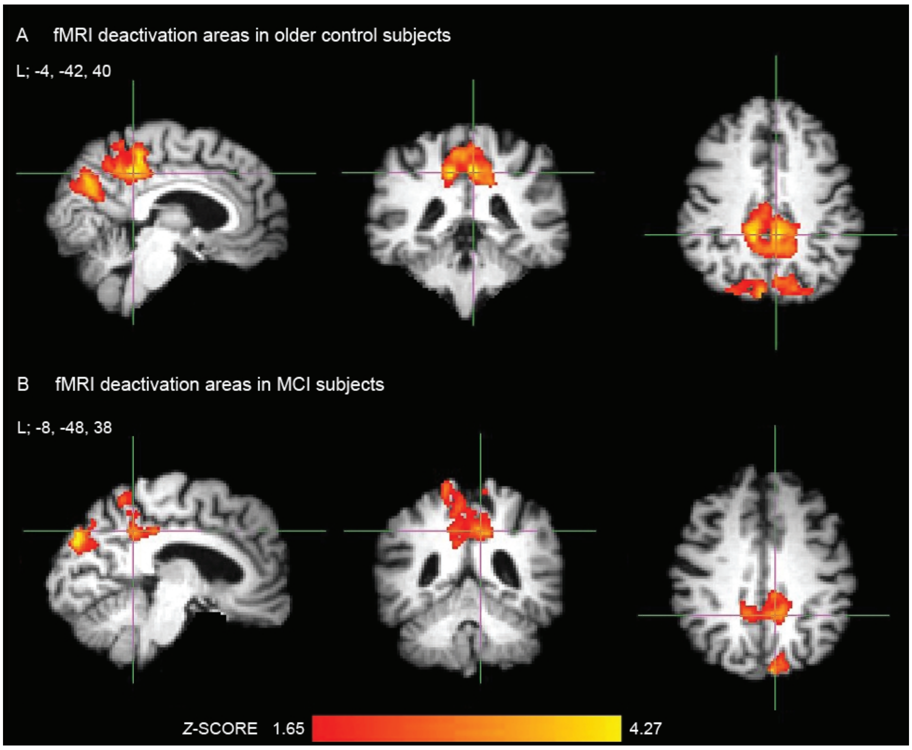

Fig. 1. (**A**) Significant bilateral precuneal and posterior cingulate deactivation areas induced during an associative memory encoding task in older control subjects; MNI coordinate $(x, y, z)$ of the crosshair, located in the left (L) posterior cingulate cortex, is −4, −42, 40. The subjects with mild cognitive impairment (**B**) demonstrated smaller areas of deactivation in corresponding posterior cortical areas, coordinate of the crosshair in the left posterior cingulate cortex is −8, −48, 38.

*between the clinical groups and within and between the study subgroups* on the posterior cingulate, retrosplenial and precuneal region of interest (ROI; see Fig. 4). Precuneus was defined as the medial aspect of the posterior parietal lobe, corresponding to the medial extent of BA 7, retrosplenial cortex to refer to BAs 29 and 30, and posterior cingulate cortex to BAs 23 and 31 [8,89].

To investigate the magnitude of differences in fMRI signal within and between the study groups, the mean percent signal change of all voxels within the posteromedial ROI was calculated using FSL Featquery. The covariance of the MMSE, CDR-SB and post-scan memory test scores with fMRI deactivation (i.e., mean percent signal change) within the posteromedial ROI was studied across all study subjects by using each of these cognitive measures as a covariate of interest. The statistical threshold utilized in all of the above data analyses was cluster-corrected for multiple comparisons at $p < 0.05$.

### 2.6. *Demographic and cognitive data analysis*

Statistical analysis of demographic, cognitive and fMRI behavioral data was conducted with SPSS 14.0 software (SPSS Inc., Chicago, IL, USA) using the non-parametric Mann-Whitney U test. The level of statistically significant differences was set at $p < 0.05$.

## 3. Results

### 3.1. *Demographic and cognitive data*

The clinical study groups OC, MCI and AD differed on several demographic and cognitive variables as presented in detail in Table 1. There were no differences in the demographic and cognitive parameters between the genetic subgroups within the clinical groups, except that the MCI e4 carriers had higher ($p = 0.04$) and the AD e4 carriers lower ($p = 0.002$) CDR-SB scores than the non-carriers.

### 3.2. *FMRI deactivation within and between the OC, MCI and AD groups*

We first examined the functional integrity of the default mode network within and between the clinical

Table 2
Areas of significant task-induced deactivation within and differential deactivation between the clinical groups

| Brain region | BA | $x$ | $y$ | $z$ | Peak Z | Peak p |
|---|---|---|---|---|---|---|
| *Within clinical groups* | | | | | | |
| *Older controls* | | | | | | |
| R posterior cingulate | 23, 31 | 12 | −44 | 42 | 4.19 | $1 \times 10^{5}$ |
| L posterior cingulate | 23, 31 | −4 | −42 | 40 | 4.65 | $2 \times 10^{6}$ |
| R precuneus | 7, 19 | 12 | −82 | 32 | 4.83 | $7 \times 10^{7}$ |
| L precuneus | 7, 19 | −16 | −68 | 30 | 4.47 | $4 \times 10^{6}$ |
| L angular gyrus | 39 | −46 | −80 | 30 | 5.10 | $2 \times 10^{7}$ |
| *Mild cognitive impairment* | | | | | | |
| L posterior cingulate | 23, 31 | −8 | −48 | 38 | 3.66 | $1 \times 10^{4}$ |
| R precuneus | 7 | 20 | −44 | 60 | 3.95 | $4 \times 10^{5}$ |
| L precuneus | 7, 19 | −10 | −88 | 32 | 4.37 | $6 \times 10^{6}$ |
| *Between clinical groups* | | | | | | |
| *Older controls > Mild cognitive impairment* | | | | | | |
| L precuneus | 7 | −16 | −68 | 32 | 3.74 | $9 \times 10^{5}$ |
| *Older controls > Alzheimer's disease* | | | | | | |
| R posterior cingulate | 23, 31 | 14 | −48 | 34 | 3.41 | $3 \times 10^{4}$ |
| L posterior cingulate | 23, 31 | −4 | −42 | 40 | 4.46 | $4 \times 10^{6}$ |
| R precuneus | 7 | 10 | −74 | 32 | 3.73 | $1 \times 10^{4}$ |
| L precuneus | 7 | −8 | −72 | 34 | 5.67 | $7 \times 10^{9}$ |
| *Mild cognitive impairment > Alzheimer's disease* | | | | | | |
| L precuneus | 7 | −10 | −76 | 42 | 4.06 | $2 \times 10^{5}$ |

Peak $Z$-scores, corresponding uncorrected p -values and MNI coordinates $(x, y, z)$ of the brain deactivation regions and corresponding Brodmann areas (BA); L = left; R = right; threshold for statistical significance $p < 0.05$, cluster-corrected.

Table 3
Areas of task-induced deactivation demonstrating significant correlation with cognitive measures across all study subjects

| Brain region | BA | $x$ | $y$ | $z$ | Peak Z | Peak p |
|---|---|---|---|---|---|---|
| *Mini-Mental State Examination* | | | | | | |
| R posterior cingulate | 23, 31 | 16 | −38 | 40 | 3.59 | $2 \times 10^{4}$ |
| L posterior cingulate | 23, 31 | −4 | −38 | 38 | 3.17 | $8 \times 10^{4}$ |
| L precuneus | 7 | −18 | −70 | 32 | 3.89 | $5 \times 10^{5}$ |
| *Clinical Dementia Rating Sum-of-Box* | | | | | | |
| R posterior cingulate | 23, 31 | 10 | −44 | 50 | −3.83 | $6 \times 10^{5}$ |
| L posterior cingulate | 23, 31 | −4 | −42 | 40 | −4.46 | $4 \times 10^{6}$ |
| R precuneus | 7 | 20 | −68 | 22 | −3.83 | $6 \times 10^{5}$ |
| L precuneus | 7 | −8 | −70 | 32 | −5.51 | $2 \times 10^{8}$ |
| *Post-scan memory test MT2* | | | | | | |
| R precuneus | 7 | 10 | −72 | 38 | 3.88 | $5 \times 10^{5}$ |
| L precuneus | 7 | −12 | −72 | 34 | 4.47 | $4 \times 10^{6}$ |

Peak $Z$-scores, corresponding uncorrected p -values and MNI coordinates $(x, y, z)$ of the brain deactivation regions and corresponding Brodmann areas (BA); L = left; R = right; threshold for statistical significance $p < 0.05$, cluster-corrected.

study groups (Table 2 and Figs 1–2A). The OC demonstrated significant fMRI task-induced deactivation bilaterally in the posterior cingulate, precuneal and left lateral parietal cortices (Fig. 1A). Subjects with MCI showed smaller areas of deactivation roughly in the same posteromedial cortical areas as the OC group (Fig. 1B). There was no significant deactivation in AD patients.

Within the posteromedial ROI of the study, differences in deactivation between the groups of OC and MCI as well as between MCI and AD patients (Table 2, Fig. 2A) were observed in the left precuneus. When contrasting the OC to AD patients, the right precuneal and posterior cingulate cortices also demonstrated differential task-induced deactivation.

### 3.3. *Correlation of fMRI deactivation with demographic and cognitive measures*

Next, we explored whether the standard clinical cognitive measures MMSE and CDR-SB were correlat-

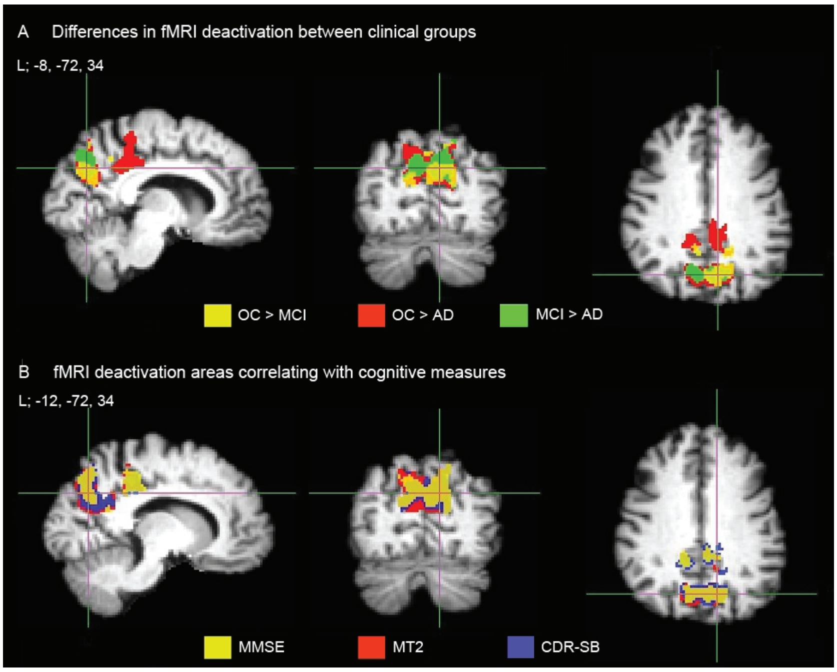

Fig. 2. (**A**) Precuneal and posterior cingulate deactivation areas where older controls (OC) demonstrated greater deactivation than subjects with mild cognitive impairment (MCI) are depicted in yellow, older controls greater than patients with Alzheimer's disease (AD) in red, and subjects with mild cognitive impairment greater than Alzheimer's patients in green. MNI coordinate $(x, y, z)$ of the crosshair, located in the left (L) precuneal cortex, is $-8$, $-72$, 34. (**B**) presents fMRI deactivation areas which showed significant positive correlation with Mini-Mental State Examination (MMSE) scores in yellow, with post-scan memory test (MT2) scores in red, and areas of significant negative correlation with Clinical Dementia Rating Sum-of-Box (CDR-SB) scores in blue. Coordinate of the crosshair, located in the left precuneal cortex, is $-12$, $-72$, 34.

ed with the fMRI deactivation pattern induced during a memory encoding task (Table 3, Fig. 2B). Within the posteromedial ROI, there was a significant positive correlation between the MMSE score and fMRI deactivation (i.e., mean percent signal change) in the posterior cingulate and left precuneal cortices, whereas the CDR-SB score demonstrated a negative correlation with fMRI deactivation bilaterally in the cingulate and precuneal regions. That is, clinically less impaired subjects in terms of higher MMSE or lower CDR-SB scores demonstrated more task-induced deactivation. Better performance on the post-scan forced-choice face-name associative recognition task (MT2) was strongly positively correlated with precuneal deactivation bilaterally, whereas we did not find any deactivation areas to correlate significantly with the post-scan memory test MT1 (face memory only).

### *3.4. FMRI deactivation within and between the genetic subgroups of OC, MCI and AD*

Finally, we investigated the effect of *APOE* e4 allele on the pattern of fMRI deactivation within each clinical group, again anatomically focusing on the posteromedial ROI of the study (Table 4, Fig. 3). Among the OC e4 non-carriers, we found significant clusters of fMRI deactivation bilaterally in the posterior cingulate and precuneal cortices. The OC e4 carriers showed smaller areas of deactivation mostly in areas similar to those observed in OC non-carriers, but not in the right posterior cingulate cortex. We found no significant deactivation areas in MCI e4 non-carriers or carriers exceeding the cluster-corrected threshold of $p < 0.05$. MCI e4 non-carriers, showed, however, a focal sub-threshold deactivation cluster (i.e., a cluster not meeting the cluster-corrected criteria for statistical significance) in the left posterior cingulate cortex (MNI coordinate of the peak location: $-14$, $-64$, 16; $Z = 3.32$, corresponding to an uncorrected $p = 0.0005$). AD e4 non-carriers showed deactivation bilaterally only in the very posterior precuneal regions, close to the parieto-occipital fissure, whereas there was no deactivation observed in AD e4 carriers.

In the direct statistical comparison between the genetic subgroups, the OC e4 non-carriers showed significantly more task-induced deactivation in the posterior cingulate and precuneal cortices than OC e4 carriers

Table 4
Areas of significant task-induced deactiation within and differential deactivation between the genetic subgroups

| Brain region | BA | x | y | z | Peak Z | Peak p |
|---|---|---|---|---|---|---|
| *Within genetic subgroups* | | | | | | |
| *Older controls APOE $\varepsilon$4-* | | | | | | |
| R posterior cingulate | 23, 31 | 14 | −32 | 38 | 3.72 | $1 \times 10^4$ |
| L posterior cingulate | 23, 31 | −4 | −42 | 40 | 4.17 | $2 \times 10^5$ |
| R precuneus | 7 | 10 | −82 | 32 | 4.24 | $1 \times 10^5$ |
| L precuneus | 7 | −14 | −68 | 32 | 4.14 | $2 \times 10^5$ |
| *Older controls APOE $\varepsilon$4+* | | | | | | |
| L posterior cingulate | 23, 31 | −10 | −36 | 56 | 4.43 | $5 \times 10^6$ |
| L precuneus | 7 | 8 | −88 | 40 | 4.16 | $2 \times 10^5$ |
| *Alzheimer's disease APOE $\varepsilon$4-* | | | | | | |
| R precuneus | 7 | 12 | −82 | 38 | 3.90 | $5 \times 10^5$ |
| L precuneus | 7 | −18 | −80 | 36 | 3.92 | $4 \times 10^5$ |
| *Between genetic subgroups* | | | | | | |
| *Older controls APOE $\varepsilon$4- $>$ $\varepsilon$4+* | | | | | | |
| R posterior cingulate | 23, 31 | 12 | −40 | 26 | 3.84 | $6 \times 10^5$ |
| R precuneus | 7 | 16 | −64 | 38 | 3.75 | $1 \times 10^4$ |
| L precuneus | 7 | −20 | −64 | 36 | 3.22 | $6 \times 10^4$ |
| *Alzheimer's disease APOE $\varepsilon$4- $>$ $\varepsilon$4+* | | | | | | |
| R precuneus | 7 | 12 | −82 | 40 | 3.83 | $1 \times 10^4$ |
| L precuneus | 7 | −20 | −78 | 30 | 3.17 | $8 \times 10^4$ |

Peak $Z$-scores, corresponding uncorrected p-values and MNI coordinates $(x, y, z)$ of the brain deactivation regions and corresponding Brodmann areas (BA); L = left; R = right; threshold for statistical significance $p <$ 0.05, cluster-corrected.

(Fig. 3A). In this study, we did not observe statistically significant differences in deactivation between the genetic subgroups of MCI but the MCI e4 carriers demonstrated a trend towards less deactivation than MCI non-carriers in the left posterior cingulate cortex (MNI coordinate of the peak location: $-10, -66, 22$; $Z = 2.70$, corresponding to an uncorrected $p = 0.004$). Similar to the findings in OC, there was more deactivation in AD e4 non-carriers than in the e4 carriers (Fig. 3A).

To further explore the magnitude of fMRI signal change within and between the groups of OC, MCI and AD, and their genetic subgroups, we extracted the mean percent signal change of all voxels within the posteromedial ROI of the study (Fig. 4). There was a clear pattern of loss of deactivation across the cognitive continuum from OC to MCI and to mild AD. The OC e4 carriers demonstrated less deactivation than non-carriers. In MCI subjects, the e4 carriers and non-carriers showed levels of deactivation intermediate between OC and AD. Interestingly, we observed evidence of paradoxical signal increase in the MCI e4 carriers and both groups of AD patients.

## 4. Discussion

Taken together, results from these studies suggest that normal fMRI task-induced deactivation pattern of the core regions of the brain default mode network is increasingly compromised over the course of AD, with significant alterations being found in subjects at risk for AD either in terms of having MCI or the *APOE* e4 risk genotype or both. Greater cognitive impairment is associated with greater disruption of the posteromedial task-induced deactivation across the continuum from healthy elderly to clinical AD patients, and even more so in the e4 carriers compared to non-carriers.

Our results in MCI and AD patients compared to OC confirm and summarize findings of previous reports that the pattern of fMRI task-induced deactivation is progressively disrupted along the advancing memory impairment related to the neurodegenerative dementia of AD type. Similar to previous [$^{18}$F]fluorodeoxyglucose PET (FDG-PET) findings [33,51,55,56,66,67,78], fMRI of the posteromedial default mode regions seems promising in separating groups of elderly controls from MCI subjects and MCI from AD patients [28,44,61–63,70,71]. Disrupted fMRI deactivation in AD patients relative to elderly controls has recently been reported even during processing repeatedly presented stimuli, which become highly familiar to healthy elderly subjects (Fig. 5) [63], not only during complex and demanding cognitive tasks.

Additionally, our findings provide clinically relevant evidence that the standard cognitive measures used in AD diagnostics such as the MMSE and CDR-SB scores

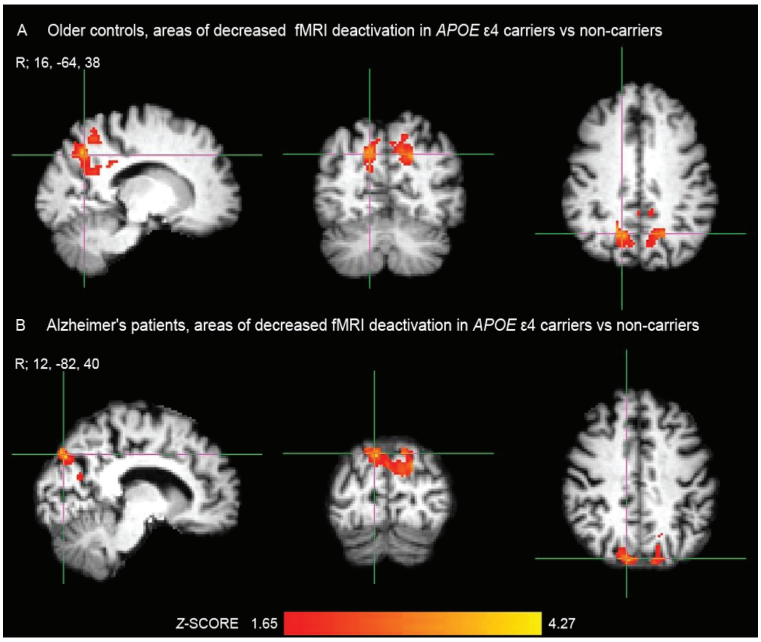

Fig. 3. (**A**) In older controls, fMRI deactivation was significantly decreased in the precuneal and posterior cingulate cortices in *APOE* e4 carriers compared to non-carriers. MNI coordinate of the crosshair ($x, y, z$), located in the right (R) precuneal cortex, is 16, −64, 38. (**B**) Similarly, in patients with Alzheimer's disease, the failure of deactivation was more pronounced in e4 carriers than in non-carriers. Coordinate of the crosshair, located in the right posterior precuneal cortex bordering to the parieto-occipital fissure, is 12, −82, 40.

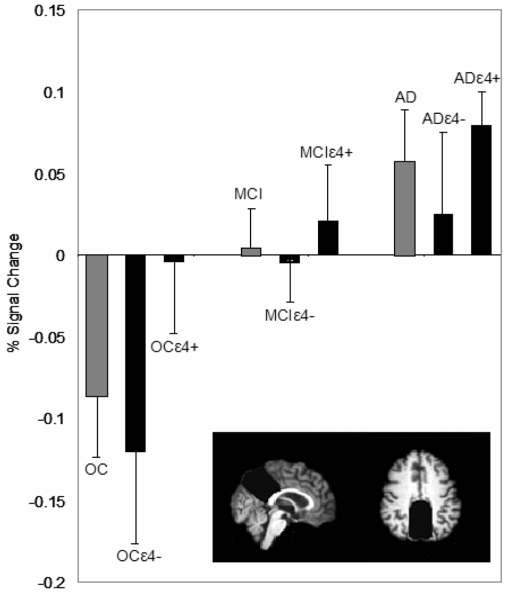

Fig. 4. Summary of the results of our recent studies presenting the mean percent signal change (and standard error of the mean) in each of the clinical groups (in gray) and their genetic subgroups (in black). The posteromedial cortical region of interest is illustrated in the right lower corner. Within older controls (OC), the e4 carriers (OCe4+) demonstrated less deactivation than non-carriers (OCe4–). Within subjects with mild cognitive impairment (MCI), the e4 carriers (MCIe4+) and non-carriers (MCIe4–) showed levels of deactivation intermediate between OC and patients with Alzheimer's disease (AD). The failure of deactivation was most pronounced in AD patients, who actually demonstrated positive fMRI activation responses in brain areas that normally deactivate in OC.

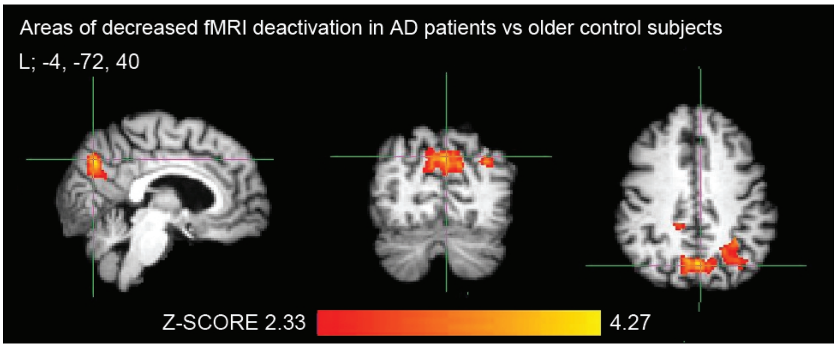

Fig. 5. Posteromedial cortical areas demonstrating deacreased fMRI task-induced deactivation during processing of repeated face-name pairs in patients with Alzheimer's disease ($n = 15$) compared to older controls ($n = 29$) [63]. MNI coordinate of the crosshair ($x, y, z$), located in the left (L) precuneus, is $-4$, $-72$, 40. Threshold for statistical significance was a cluster-corrected $p < 0.5$.

are correlated with posterior cortical deactivation induced during an associative memory encoding task. In other words, clinically less impaired subjects in terms of higher MMSE or lower CDR-SB scores demonstrated greater task-induced deactivation in the posteromedial regions of the default network, and vice versa. The brain areas that showed significant correlation with MMSE and CDR-SB scores (Fig. 2B) were very similar to the areas demonstrating differential deactivation in comparisons between clinical groups (Fig. 2A), which is not surprising as both cognitive measures are commonly used in diagnostics of MCI and AD. Interestingly, task-induced deactivation was also positively correlated with performance on the post-scan memory test for the fMRI face-name encoding paradigm, a parameter that is not related to the study subjects' clinical diagnostics. This finding suggests that better associative memory encoding performance during imaging – which is then seen as better post-scan memory test performance – is related to more task-induced deactivation. This is consonant with recent studies in healthy young and older adults [13,25,50,57,91] demonstrating that the ability to suspend default mode activity during goal-directed cognitive tasks, i.e. to reallocate neurocognitive resources to those brain regions optimal for the task performance, may be critical for successful cognitive performance. It is likely that the pathologically affected MCI or AD brain [75,86] is no longer capable of "turning off" the areas of the default mode network during focused cognitive processing in order to optimally recruit other networks such as the hippocampal memory network or frontoparietal attentional network for task performance [18,49,50,91].

This study also provides evidence that the *APOE* e4 risk genotype has an additional effect on the fMRI deactivation pattern, such that in each of the study groups the e4 carriers demonstrated a more impaired deactivation pattern than the non-carriers. The finding of disrupted posteromedial cortical deactivation in the cognitively normal e4 carriers versus non-carriers is consistent with the results of previous FDG-PET studies demonstrating impaired glucose metabolism in corresponding brain regions [67,78], and also with recent fMRI deactivation studies in cognitively intact middle-aged and elderly e4 carriers and non-carriers [58,64]. We also found that the e4 allele still continues to modulate the fMRI response pattern in clinical AD such that the presence of *APOE*e4 was associated with notable failure of deactivation, or actually with paradoxical fMRI activation response in the posteromedial cortices. This finding of paradoxical, or "reversed" activation is similar to the study by Lustig et al. [44], but has not been reported in all fMRI deactivation studies of AD. Interestingly, in this study we observed evidence of paradoxical activation also among the MCI e4 carriers, not yet meeting clinical criteria for dementia. In fact, the posteromedial cortical fMRI deactivation pattern of the MCI e4 carriers being in a "double risk" for AD was very similar to the response pattern observed in the AD patients. This observation needs to be confirmed in future studies and also using more specific regions of interest, but is very interesting, also given the recent results by Petrella et al. [61] which suggested that loss of posteromedial cortical deactivation may have diagnostic and prognostic value – that is, potentially aid in identifying the MCI individuals at the greatest future risk for progressive cognitive decline and conversion to clinical AD.

Posterior cortical regions of the default mode network not only overlap with the pattern of FDG-PET hypometabolism but also with the distribution of the fibrillar amyloid-beta (A$\beta$) deposition in AD [7]. Neuritic

A$\beta$ plaques are, in addition to neurofibrillary tangles and neuronal and synaptic loss, the pathological hallmark of AD [4,24,36,74,75,86]. Recent PET studies using a tracer called [$^{11}$C]Pittsburgh Compound B, or PIB, labeling A$\beta$ plaques [17,40] have suggested that the posteromedial cortical areas of high default mode activity may be among the earliest sites of A$\beta$ pathology in AD [52]. Thus, functional (or structural) imaging studies focusing on this region are pathobiologically very relevant when searching for potential early markers of prodromal AD.

In MCI subjects, and in clinical AD patients in particular, the posteromedial cortical alterations observed in the fMRI deactivation studies [28,44,61–63,70,71] may also reflect remote effects of the medial temporal lobe pathology and atrophy. The medial temporal lobe, which is thought be responsible for the episodic memory deficits observed in amnestic MCI and AD and is known to present the earliest neurofibrillary changes and neuronal and synapse loss, is strongly connected to the posteromedial nodes of the default network [4, 23,34,35,42,43,85]. This idea of functional alterations due to disconnection between the medial temporal and posteromedial cortices has been investigated in a study by Meguro et al. [48] using rhinal cortical lesions in non-human primates as well as in a post-mortem study in AD patients [5], both of which reported hypometabolism and hypoperfusion in the posterior cingulate and parietal cortices due to the medial temporal lobe lesions. Studies by Reiman et al. [68] and Kogure et al. [41], however, found reduced posteromedial cortical metabolism before measurable medial temporal lobe volumetric or metabolic decline, which again suggests a promising role for functional imaging of the posteromedial cortices in early diagnosis of AD and evaluation of subjects at risk for AD.

It is possible that structural atrophy of the underlying brain regions (not only remote medial temporal regions) may explain some of the findings of altered deactivation in the default mode regions found in this study as well as in other related functional imaging studies in AD [28,44,61–63,70,71]. Some structural MRI studies, but not all, have reported gray matter atrophy within the posteromedial cortices already at the stage of MCI, particularly in subjects who later progressed to clinical AD compared to those who remained cognitively stable [1,10,32,73,87,92]. Regarding the *APOE*e4 carrier genotype, elderly e4 carriers seem to suffer medial temporal lobe atrophy but not any excessive global brain atrophy [14,22,79]. Optimally, however, prodromal AD would be detected before prominent irreversible structural atrophy – for example using functional imaging methods – at a point when disease-modifying therapies still might be able to prevent the progression to clinical dementia. Future studies are needed to evaluate the temporal cascade between posteromedial cortical atrophy and altered fMRI deactivation in the earliest stages of AD.

The "default mode" of the human brain – the function of which is evaluated in this study using task-induced deactivation fMR imaging as an estimate of the default mode network – was originally identified by its consistent activity increases during passive task states (or, baseline rest conditions) as compared to a wide range of active goal-directed tasks [6,30,46,65, 76]. Regions of the default network show high resting glucose metabolism and high regional blood flow relative to other brain regions [6,51,65]. Currently, the brain default network is considered a large-scale neural system of it's own, with suggested cognitive functions such as memory recollection, theory of mind, metacognition, affect appraisal etc. [6,8,11,38,69]. Altogether, the human brain default mode network is still a relatively new concept, which should be evaluated critically and there is, in fact, lot's of critic regarding this concept as well [53,83]. It is also worth noting that task-related BOLD fMRI is always and only a relative measure investigating the difference between two cognitive conditions – in other words, there is not an absolute quantitative zero baseline from which BOLD signal would vary up or down.

A more recent approach – the functional connectivity MRI (fcMRI) – identifies brain systems via intrinsic functional (activity) correlations and is very informative because it provides a means to assess interacting brain regions during an awake rest state in a manner that is independent of task-induced deactivation imaging [12,20,26,27]. Recent fcMRI studies in healthy young subjects have demonstrated the consistency of the resting state networks in the human brain and have also corroborated the findings of altered task-induced deactivation in MCI and AD relative to controls in a complementary way [9,80,84,90,95]. As an example, functional connectivity between the posteromedial and medial temporal cortices has been reported to be impaired even in MCI subjects relative to healthy elderly controls [80,95]. Resting state fcMRI between the medial temporal and posteromedial cortices has also recently been demonstrated to reflect underlying structural connectivity as revealed by diffusion tensor imaging [29].

Future studies investigating the relations between the fMRI task-induced activation / deactivation imaging and resting fcMRI, perhaps also incorporating perfusion MRI imaging, will advance our knowledge of the default network and it's pathophysiological alterations during the long prodromal phase of AD. Furthermore, multimodal imaging studies combining task-related fMRI and other MRI techniques with molecular imaging such as PIB-PET amyloid imaging will further expand our understanding of the role of the amyloid pathology and impaired default mode network function in the pathogenesis and cognitive symptomatology of AD.

## Acknowledgments

This study was supported by Academy of Finland grants #108188 and #214050 as well as the following grants: NINDS K23-NS02189, NIA R01 AG027435, NIA PO1-AG04953, and NIA P50-AG00513421. We thank the staff of the Massachusetts General Hospital Gerontology Research Unit and Brigham and Women's Hospital Memory Disorders Unit Clinical Research for assistance with subject recruitment, evaluation, and data management, as well as Mary Foley, Larry White, and the Athinoula A. Martinos Center staff for assistance with MRI data collection. We express special gratitude to the subjects who participated in this study.
